# Effects of Depot Medroxyprogesterone Acetate, Copper Intrauterine Devices, and Levonorgestrel Implants on Early HIV Disease Progression

**DOI:** 10.1089/aid.2020.0015

**Published:** 2020-07-30

**Authors:** Charles S. Morrison, G. Justus Hofmeyr, Katherine K. Thomas, Helen Rees, Neena Philip, Thesla Palanee-Phillips, Kavita Nanda, Gonasagrie Nair, Maricianah Onono, Timothy D. Mastro, Maggie Lind, Renee Heffron, Vinodh Edward, Jen Deese, Mags Beksinska, Ivana Beesham, Jeffrey S.A. Stringer, Jared M. Baeten, Khatija Ahmed

**Affiliations:** ^1^ FHI 360, Durham, North Carolina, USA.; ^2^ Effective Care Research Unit, Department of Obstetrics and Gynaecology, University of Witwatersrand/Fort Hare/Walter Sisulu, East London, South Africa.; ^3^ Department of Obstetrics and Gynaecology, University of Botswana, Gaborone, Botswana.; ^4^ Department of Global Health, University of Washington, Seattle, USA.; ^5^ Wits Reproductive Health and HIV Institute (Wits RHI), University of Witswatersrand, Johannesburg, South Africa.; ^6^ Mailman School of Public Health, ICAP at Columbia University, New York, USA.; ^7^ Emavundleni Research Center, Capetown, South Africa.; ^8^ KEMRI-RCTP Study Center, Kisumu, Kenya.; ^9^ The Aurum Institute, Johannesburg, South Africa.; ^10^ Department of Environmental Health Sciences, Yale School of Public Health, New Haven, Connecticut, USA.; ^11^ Faculty of Health Sciences, School of Pathology, University of the Witwatersrand, Johannesburg, South Africa.; ^12^ MatCH Research Unit (MRU), Faculty of Health Sciences, Department of Obstetrics and Gynecology, University of the Witwatersrand, Durban, South Africa.; ^13^ Department of Obstetrics and Gynecology, University of North Carolina School of Medicine, Chapel Hill, North Carolina, USA.; ^14^ Setshaba Research Center, Soshanguve, South Africa.

**Keywords:** hormonal contraception, DMPA, intrauterine device, implants, HIV, disease progression, viral suppression

## Abstract

Limited data exist on the effects of contraceptives on HIV disease progression. We studied the association between intramuscular injectable depot medroxyprogesterone acetate (DMPA-IM), the copper intrauterine device (IUD), and the levonorgestrel (LNG) implant on markers of HIV disease progression at the time of HIV detection and 3 months postdetection and time from detection to CD4 count <350 cells/mm^3^. Among women initiating antiretroviral therapy (ART), we studied the effect of contraceptive group on time from ART initiation to viral load (VL) <40 copies/mL. We included women 16–35 years randomized to DMPA-IM, copper IUD, or LNG implant with incident HIV infection during the Evidence for Contraceptive Options and HIV Outcomes (ECHO) trial (*n* = 382). We analyzed HIV VL and CD4 cell count according to participants' randomized method and also conducted a “continuous use” analysis that excluded follow-up time after method discontinuation. We used adjusted linear models to compare mean VL and CD4 cell levels by contraceptive group up to the time of ART initiation. We compared time from HIV detection to CD4 count <350 cells/mm^3^ and, following ART initiation, time to viral suppression (VL <40 copies/mL) using Cox proportional hazards models. At HIV detection, women allocated to DMPA-IM had lower VL relative to copper IUD (−0.28 log_10_ copies/mL; 95% confidence interval [CI]: −0.55 to −0.01) and LNG implant (−0.27, CI: −0.55 to 0.02) and higher mean CD4 than copper IUD users by 66 cells/mm^3^ (CI: 11–121). In continuous use analyses women allocated to DMPA-IM progressed to CD4 < 350 cells/mm^3^ slower than copper IUD users (hazard ratio [HR] = 0.6, CI: 0.3–1.1), whereas copper IUD users progressed faster than LNG implant users (HR = 1.8, CI: 1.0–3.3). Time to viral suppression was faster for DMPA-IM than copper IUD (HR = 1.5, CI: 1.0–2.3) and LNG implant 1.4 (CI: 0.9–2.2) users. We found no evidence of more rapid early HIV disease progression among women using DMPA-IM than among women using copper IUD or LNG implant. Our finding of more rapid progression among copper IUD compared with DMPA-IM users should be interpreted cautiously.

## Introduction

There are ∼18 million women living with HIV, a majority live in sub-Saharan Africa, and African women make up over a third of the 1.6 million new adult infections worldwide in 2017.^1^. While contraceptive use vastly improves maternal and child morbidity and mortality, approximately half of African women not desiring pregnancy have an unmet need for modern contraception.^2^ Intramuscular injectable depot medroxyprogesterone acetate (DMPA-IM) is the predominant contraceptive used in much of sub-Saharan Africa.

The potential immunosuppressive effects of DMPA-IM may influence early markers of HIV disease progression.^3,4^ One trial in Zambia among women with established infection found that HIV progressed more quickly among women randomized to hormonal contraceptives (HC), including DMPA-IM, compared with women randomized to copper intrauterine device (copper IUD).^5^ In this study, however, participant retention was suboptimal and women often discontinued their randomized method. Multiple cohort studies have found no association between DMPA-IM use and HIV disease progression,^6,7^ although few have been designed specifically to evaluate this association. Few data exist on the effect of contraceptive implants on HIV disease progression.^7^

Hormonal contraception may promote HIV disease progression by modifying either the initial infection (primary effect) or subsequent progression (secondary effect). An observational study found accelerated changes in markers of HIV disease in women who acquired infection while using DMPA-IM,^8^ possibly mediated by initial infection with multiple HIV strains.^8,9^ The Zambian trial—in which HIV disease progression was not a planned endpoint—suggested a secondary effect, as randomization to contraceptive method took place after HIV infection.

The Evidence for Contraceptive Options and HIV Outcomes (ECHO) trial was an open-label randomized trial comparing incident HIV infection among women randomly allocated to one of the three trial methods: DMPA-IM, copper IUD, or levonorgestrel (LNG) implant.^10^ The trial found no substantial difference in HIV acquisition among women using the three contraceptive methods.^10^

The ECHO trial protocol (ClinicalTrials.gov number NCT02550067)^11^ included a planned analysis of disease progression by contraceptive method among women acquiring HIV during the trial. The objective of the present analysis was to determine the relative impact of the three contraceptive methods on HIV disease progression as measured by viral load (VL) and CD4 cell count at the HIV detection and 3-month postdetection visits, time to CD4 < 350 cells (before starting antiretroviral therapy [ART]) and, following ART initiation, time to viral suppression (<40 copies/mL).

## Materials and Methods

### Study design and procedures

The ECHO trial methods are reported elsewhere.^10^ Briefly, we conducted a randomized, multicenter, open-label trial at 12 research sites: 9 in South Africa and 1 each in Eswatini, Kenya, and Zambia. We enrolled nonpregnant, HIV-seronegative women age 16–35 years who desired effective contraception, had no medical contraindications to the trial contraceptive methods, agreed to use the assigned contraceptive for 18 months, and reported not using injectable, intrauterine, or implantable contraception for the previous 6 months. Ethics Review Committees at each study site, FHI 360, and the World Health Organization (WHO) approved the study protocol.

At enrollment into the ECHO trial, women were randomly assigned (1:1:1) to DMPA-IM, copper IUD, or LNG implant stratified by site. Participants received an injection of 150 mg/mL DMPA-IM (Depo Provera; Pfizer, Puurs, Belgium) at enrollment and then every 3 months, or a copper IUD (Optima TCu380A; Injeflex, Sao Paolo, Brazil) or LNG implant (Jadelle; Bayer, Turku, Finland) at enrollment. Women returned for scheduled follow-up visits every 3 months up to 18 months for visits that included HIV serological testing, contraceptive counseling, and safety monitoring. At enrollment and the HIV detection visit we tested for sexually transmitted infections (STIs; *Chlamydia trachomatis*, *Neisseria gonorrhoeae*, and HSV-2) and provided treatment for curable STIs.^10^ At other follow-up visits we provided syndromic STI management.^12^ Additionally, we provided comprehensive HIV risk reduction counseling, including participant and partner HIV testing and management, condoms, and pre-exposure prophylaxis as it became part of the various national standards of care.^10^

We defined incident HIV infection using a standard algorithm, as previously described,^10^ and an endpoint committee adjudicated seroconversion status. For women testing HIV seropositive, we analyzed archived plasma samples from the enrollment visit using HIV RNA polymerase chain reaction and identified those with detectable HIV RNA as pre-enrollment infections. We linked women who acquired HIV to local care and treatment. Each study site had written standard operating procedures (SOP) in place for referral to HIV care and treatment clinics providing ART assessment, initiation, and management; and care and support services. Referrals and care and treatment services for HIV were independent of contraceptive method use.

Women continued routine study follow-up after HIV detection per study schedule with no further HIV serologic testing or related pre- and posttest counseling.^10,11^ Women with incident HIV infection continued their study contraceptive methods unless otherwise contraindicated or they chose to discontinue. Plasma HIV RNA and CD4 testing were conducted at the HIV detection visit and quarterly thereafter.

### Disease progression analysis population

There were 412 women with HIV detected during the ECHO trial. We excluded 15 women who started ART before HIV detection in ECHO and, in our primary analysis, an additional 15 women who were HIV-seronegative but later found to be HIV infected at enrollment. Thus, our primary analyses are limited to the 382 women HIV uninfected at enrollment and ART naive at HIV detection. Because the numbers providing information on secondary effects of contraception on HIV progression (the 15 women HIV infected at enrollment) were too small for meaningful statistical analysis, we present a separate descriptive analysis for this group of women.

### Outcomes

The designated primary outcome *a priori* was HIV VL closest to 3 months post-HIV detection (when viral setpoint was expected to be attained) among women remaining ART naive. However, because few women were ART naive at this time point, this outcome was less informative than other endpoints, including HIV VL and CD4 cell counts at the HIV detection visit, time from HIV detection (before ART initiation) to CD4 < 350 cells/mm^3^ and, among women initiating ART, time from ART initiation to viral suppression (VL <40 copies/mL).

### Statistical analysis

#### VL and CD4 at HIV detection and 3-month visits

To compare disease progression markers by contraceptive method, we compared HIV-1 VLs and CD4 counts at HIV detection and 3 months post-HIV detection, by randomized method, until women reported beginning ART, at which point their data were censored. We estimated pairwise differences in the mean HIV VL (or CD4 count) for each group vs. each other group using linear models. All models were adjusted for baseline confounders at ECHO trial enrollment defined as covariates that altered the estimated difference between any of the groups by 10% or more in continuous use analysis. Covariates considered for adjustment were age, HSV-2 serostatus, *C. trachomatis*, and *N. gonorrhoeae* status and pregnancy. We considered effect modification by STI status (defined as positive for *C. trachomatis*, *N. gonorrhoeae*, or HSV-2 at enrollment) by testing an interaction term between STI status and group.

### Analysis of time to CD4 < 350 cells/mm^3^

We analyzed time to CD4 < 350 cells using Cox models to estimate the three pairwise hazard ratios (HRs) and 95% CIs comparing each group versus each other group and adjusting for the baseline variables age, *C. trachomatis*/*N. gonorrhoeae*, and HSV-2 serostatus.

### Analysis of time from ART initiation to viral suppression

To examine ART effectiveness by contraceptive method, we compared time with viral suppression using Cox regression to estimate HRs between groups. Time to viral suppression was defined as months from the first study visit at which ART use was reported, to the first visit with HIV VL <40 copies/mL. If HIV VL was <40 copies/mL at the first study visit at which ART use was reported, the time to event was assigned as 0.1 months.

All analyses were done both “as randomized” and limited to time with “continuous use.” The as-randomized analysis used the randomly assigned contraceptive method regardless of actual method use at a later time. We defined “continuous use” of a method to mean the woman started using her randomized method at enrollment (or within 28 days for copper IUD), and had no discontinuations up to the day of the blood draw. Discontinuations were defined as >17 weeks elapsing between DMPA-IM injections, removal of a user's LNG implant without reinsertion on the same day; or expulsion/removal of a copper IUD without reinsertion within 28 days. All analyses were performed using R version 3.5.3.

## Results

### Study population

For the 382 women in the primary analysis, 133 women had been randomized to DMPA-IM, 114 to LNG implant, and 135 to copper IUD. The median time from enrollment to seroconversion was 280 days. At enrollment, most women were young (71% ≤ 24 years), parous (94%), had never been married (93%), and were not living with a partner (87%) (Table 1). Twelve percent reported >1 sex partner in the 3 months before enrollment and about half (48%) reported no condom use with their last sexual act. STIs were highly prevalent: 25% had *C. trachomatis*, 10% had *N. gonorrhoeae*, and 56% were HSV-2 infected. Participant characteristics were similar among women randomized to the three contraceptive groups.

**Table 1.** Characteristics of HIV-Uninfected Participants at ECHO Enrollment and at HIV Detection Visit Expressed as Number (Percent) or Median (Interquartile Range), by Group Allocation (*n* = 382)

	Total (*n* = 382)		DMPA-IM (*n* = 133)		Copper IUD (*n* = 135)		LNG implant (*n* = 114)		p^a^
Characteristics at ECHO enrollment									
24 Years of age or less (*N*, %)	270	70.7%	94	70.7%	98	72.6%	78	68.4%	.771
BMI obese (≥30) (*N*, %)	89	23.3%	34	25.6%	25	18.5%	30	26.3%	.261
Nulliparous (*N*, %)	24	6.3%	13	9.8%	8	5.9%	3	2.6%	.063
Ever married (*N*, %)	26	6.8%	9	6.8%	6	4.4%	11	9.6%	.267
Living with partner (*N*, %)	50	13.1%	15	11.3%	17	12.6%	18	15.8%	.565
Earns own income (*N*, %)	72	18.8%	21	15.8%	30	22.2%	21	18.4%	.400
Sex partners past 3 months (mean, SD)	1.1	0.3	1.1	0.4	1.1	0.3	1.1	0.4	.549
Coital acts past 3 months (mean, SD)	11.6	12.5	11.4	12	10.7	11.2	12.9	14.5	.382
Condomless sex ever past 3 months (*N*, %)	290	75.9%	106	79.7%	98	72.6%	86	75.4%	.392
Condom use last vaginal sex (*N*, %)	198	51.8%	66	49.6%	71	52.6%	61	53.5%	.811
Sex for money or gifts past 3 months (*N*, %)	6	1.6%	2	1.5%	1	0.7%	3	2.6%	.488
No previous contraceptive use (*N*, %)	17	4.5%	7	5.3%	6	4.4%	4	3.5%	.801
STIs prevalence (*N*, %)									
*Chlamydia trachomatis*	97	25.4%	32	24.1%	31	23.0%	34	29.8%	.395
*Neisseria gonorrhoeae*	38	9.9%	9	6.8%	15	11.1%	14	12.3%	.293
HSV-2	213	56.2%	79	60.3%	75	56.0%	59	51.8%	.404
Characteristics at HIV detection visit									
Pregnant	14	3.7%	4	3.0%	3	2.2%	7	6.1%	.230
PrEP use^b^	2	0.5%	2	1.5%	0	0.0%	0	0.0%	.156
Sex partners past 3 months (mean, SD)	1.1	0.4	1	0.3	1.1	0.5	1.1	0.3	.422
Coital acts past 3 months (mean, SD)	13.4	14.5	10.5	12.3	13.2	13.3	16.9	17.2	.001
Condomless sex ever past 3 months	296	77.5%	100	75.2%	101	74.8%	95	83.3%	.192
Condom use last vaginal sex	169	44.8%	62	48.1%	63	46.7%	44	38.9%	.314
Sex for money or gifts past 3 months	2	0.5%	1	0.8%	0	0.0%	1	0.9%	.567
STIs prevalence									
*C. trachomatis*	80	20.9%	26	19.5%	34	25.2%	20	17.5%	.311
*N. gonorrhoeae*	58	15.2%	13	9.8%	21	15.6%	24	21.1%	.044
HSV-2	281	73.6%	99	74.4%	105	77.8%	77	67.5%	.145

Table excludes women who started ART before HIV detection.

^a^ Categorical variables tested using chi-squared tests for independence and continuous variables tested with a one-factor ANOVA.

^b^ Since the last visit PrEP was used.

ART, antiretroviral therapy; DMPA-IM, intramuscular injectable depot medroxyprogesterone acetate; IUD, intrauterine device; LNG, levonorgestrel; PrEP, pre-exposure prophylaxis; STI, sexually transmitted infection.

At the HIV detection visit, 10% of participants reported >1 sex partner in the prior 3 months and 55% reported no condom use with the last sexual act (Table 1). STI prevalences were again very high with 21% having *C. trachomatis*, 15% *N. gonorrhoeae*, and 74% were HSV-2 infected. Participant characteristics at the HIV detection visit were relatively similar among the contraceptive groups, except that the DMPA-IM seroconverters reported fewer coital acts in the previous 3 months and were less likely to be infected with *N. gonorrhoeae*.

### Follow-up time contributed

Among the 382 participants in the as-randomized analysis, 331 contributed to the continuous use analysis (80%, 87%, and 94% of DMPA-IM, copper IUD, and LNG implant users, respectively) (Table 2). Only 31% of the as-randomized population and 28% of the continuous use population contributed subsequent pre-ART follow-up data (3 months post-HIV detection and beyond); most other women had already started ART or had completed the study.

**Table 2.** Summary of HIV Seroconverter Data Available by Months Since HIV Detection Visit

	HIV detection visit	M3,* n *(%)	M6,* n *(%)	M9,* n *(%)	M12,* n *(%)	M15,* n *(%)
As-randomized (*n* = 382)^a^						
*N* (%) attended visit						
DMPA-IM	133	34.5	18.9	10.0	9.8	6.3
Copper IUD	135	36.1	19.3	14.3	9.4	9.1
LNG implant	114	39.6	17.4	15.3	15.2	13.6
Consistent-use (*n* = 331)						
*N* (%) attended visit						
DMPA-IM	106	23.1	9.3	6.8	2.4	7.7
Copper IUD	118	34.4	14.7	11.8	6.9	10.0
LNG implant	107	41.7	18.1	15.5	15.6	14.3

^a^ Excludes women infected at baseline and visits following ART initiation.

### Effect of contraceptives on measures of HIV VL

In the as-randomized analysis DMPA-IM users at the HIV detection visit had lower mean VL than copper IUD users [estimated differences: −0.28 log_10_ copies/mL (95% CI: −0.55 to −0.01)]. There was a similar magnitude of difference in VL between DMPA-IM and LNG implant users although the difference was not statistically significant [−0.27 log_10_ copies/mL (CI: −0.55 to 0.02)] (Table 3). VLs were similar in copper IUD compared with LNG implant users [difference 0.01 log_10_ copies/mL (CI −0.27 to 0.29)]. At 3 months post HIV detection, no statistically significant differences in VL were observed between the contraceptive groups although many participants had already been censored for starting ART and thus the sample size was substantially smaller. In the continuous use analysis, VL differences among contraceptive groups were similar to the as-randomized analyses at both time points, but were not statistically significant.

**Table 3.** Early Relative Effects of Contraception on Measures of HIV Viral Load and CD4 Cell Count for As-Randomized and Continuous Use Analyses Among Women Uninfected at ECHO Enrollment

	DMPA-IM	Copper IUD	LNG implant	Adjusted mean difference (95% CI)^*^		
	Mean (SD)	Mean (SD)	Mean (SD)	DMPA versus IUD^a^	DMPA versus LNG	IUD versus LNG
As randomized population						
VL (log_10_ copies/mL)						
VL: means at HIV detection visit (*n* = 381)^a^	4.25 (0.10)	4.53 (0.10)	4.52 (0.11)	**−0.28 (−0.55 to −0.01)**	**−**0.27 (**−**0.55 to 0.02)	0.01 (**−**0.27 to 0.29)
VL: means at 3 months (set point) (*n* = 114)^b^	3.98 (0.20)	3.86 (0.22)	3.86 (0.22)	0.12 (**−**0.43 to 0.67)	0.13 (**−**0.42 to 0.67)	0.00 (**−**0.54 to 0.53)
CD4 count						
CD4: means at HIV detection visit (*n* = 381)^a^	608 (25)	542 (25)	592 (26)	**66 (11** to **121)**	16 (**−**41 to 74)	**−**49 (**−**107 to 8)
CD4: means at 3 months (set point) (*n* = 114)^b^	538 (45)	559 (46)	582 (43)	**−**21 (**−**121 to 79)	**−**44 (**−**146 to 57)	**−**23 (**−**121 to 74)
Continuous use population						
VL (log_10_ copies/mL)						
VL: means at HIV detection visit (*n* = 331)^a^	4.26 (0.11)	4.52 (0.11)	4.51 (0.11)	**−**0.26 (**−**0.56 to 0.04)	**−**0.25 (**−**0.56 to 0.06)	0.01 (**−**0.29 to 0.31)
VL: means at 3 months (set point) (*n* = 92)^b^	4.10 (0.27)	4.03 (0.23)	3.85 (0.22)	0.07 (**−**0.59 to 0.74)	0.25 (**−**0.40 to 0.90)	0.18 (**−**0.39 to 0.75)
CD4 count						
CD4: means at HIV detection visit (*n* = 331)^a^	607 (28)	539 (26)	601 (27)	**68 (8 to 128)**	7 (**−**55 to 69)	**−**61 (**−**121 to **−**1)
CD4: means at 3 months (set point) (*n* = 92)^b^	550 (59)	569 (50)	603 (46)	**−**20 (**−**141 to 102)	**−**53 (**−**174 to 67)	**−**34 (**−**138 to 70)

*Bolded* values are statistically significant at *p* < .05.

^a^ At the HIV detection visit no adjustments were made for VL measures; CD4 measures were adjusted for the following baseline variables: presence of either *C. trachomatis* or *N. gonorrhoeae*, and HSV-2 serology.

^b^ At the 3-month visit (set point) VL measures were adjusted for baseline *C. trachomatis* or *N. gonorrhoeae*; CD4 measures were adjusted for the following baseline variables: age, presence of either *C. trachomatis* or *N. gonorrhoeae*, and HSV-2 serology.

DMPA, depot medroxyprogesterone acetate; VL, viral load.

### Effect of contraceptives on CD4 cell levels

In the as-randomized analysis at HIV detection, DMPA-IM users had higher CD4 cell counts than copper IUD users [difference: 66 cells/mm^3^ (95% CI: 11 to 121)] but no difference compared with LNG implant users [difference: 16 (CI: −41 to 74)]. We observed no statistically significant difference in CD4 counts between copper IUD users and LNG implant users [difference: −49 cells/mm^3^ (95% CI: −107 to 8)] (Table 3). At 3 months, no significant differences in CD4 counts among contraceptive groups were seen. In the continuous use, analysis results were similar to the as-randomized analysis for DMPA-IM compared with copper IUD [difference: 68 cells/mm^3^ (95% CI: 8–128)], with CD4 levels higher for DMPA-IM users at HIV detection, whereas no difference was seen between DMPA-IM and LNG implant users. Copper IUD users had lower CD4 counts than LNG implant users [difference: −61 cells/mm^3^ (95% CI: −121 to −1)]. Differences in CD4 counts between groups in the continuous use analysis were not statistically significant at 3 months post-HIV detection.

### Effect of contraceptive method on time to CD4 < 350 cells/mm^3^

The incidence rates for time from HIV detection to CD4 decline <350 cells/mm^3^ in the as-randomized population varied from a low of 1.2 (0.7, 1.8) per woman-year (wy) for LNG implant to a high of 2.5 (1.7, 3.4) per wy for copper IUD (Table 4). HRs ranged from 0.6 (0.4, 1.1) for DMPA-IM versus copper IUD to 1.6 (0.9, 2.9) for IUD compared with LNG implant. HRs were similar for the continuous use analysis and ranged from 0.6 (0.3, 1.1) for DMPA-IM versus copper IUD to 1.8 (1.0, 3.3) for the copper IUD versus LNG implant.

**Table 4.** Relative Effects of Contraceptive Method on Time to CD4 Count <350: Rates and Hazard Ratios Among Women Uninfected at ECHO Enrollment and Not Using Antiretroviral Therapy

N events/woman-years, rate (95% CI)^a^				HR (95% CI)					
Population	DMPA	IUD	LNG	DMPA versus IUD^b^	p	DMPA versus LNG^b^	p	IUD versus LNG^b^	p
As randomized (*n* = 382)	24/18.8, 1.3 (0.8–1.8)	33/13.0, 2.5 (1.7–3.4)	20/16.0, 1.2 (0.7–1.8)	0.6 (0.4–1.1)	.087	1.0 (0.6–1.9)	.925	1.6 (0.9–2.9)	.089
Continuous use (*n* = 331)	17/8.4, 2.0 (1.1–3.0)	30/9.6, 3.1 (2.0–4.2)	19/16.1, 1.2 (0.7–1.7)	0.6 (0.3–1.1)	.125	1.2 (0.6–2.3)	.674	1.8 (1.0–3.3)	.044

HRs were adjusted for the following baseline variables: age, presence of either *C. trachomatis* or GC, and HSV-2 serology. Women initiating ART were censored at the last blood draw before ART initiation.

^a^ Per woman-year.

^b^ Comparison group.

HR, hazard ratio.

### Effect of contraceptive method on time to viral suppression following ART initiation

In time to viral suppression analysis, 265 women who started ART contributed to the as-randomized analysis and 213 women contributed to the continuous use analysis. The incidence of viral suppression among women in the as-randomized analysis population ranged from 1.8 (1.3, 2.2) per woman year for DMPA-IM to 1.4 (1.0, 1.8) for both copper IUD and LNG implant groups (Table 5). HRs were 1.3 (0.9, 2.0) for DMPA-IM versus copper IUD, 1.2 (0.8, 1.8) for DMPA-IM versus LNG implant, and 1.0 (0.6, 1.4) for LNG implant versus the copper IUD group. Incidence rates for the continuous use analysis population were quite similar to the as-randomized population. However, differences between groups were more pronounced for the continuous use analysis population with HRs of 1.5 (1.0, 2.3) for DMPA-IM versus copper IUD, 1.4 (0.9, 2.2) for DMPA-IM versus LNG implant, and 1.0 (0.6, 1.5) for copper IUD versus LNG implant.

**Table 5.** Relative Time from Antiretroviral Therapy Initiation to Viral Suppression (Viral Load <40 Copies/mL) Among Women Taking Antiretroviral Therapy and HIV Uninfected at ECHO Enrollment: Incidence Rates and Hazard Ratios

N events/woman-years, rate*^a^*(95% CI)				HR (95% CI)^b^					
Population	DMPA	IUD	LNG	DMPA versus IUD^c^	p	DMPA versus LNG^c^	p	IUD versus LNG^c^	p
As randomized (*n* = 265)	66/37.5, 1.8 (1.3–2.2)	47/33.5, 1.4 (1.0–1.8)	46/32.2, 1.4 (1.0–1.8)	1.3 (0.9–2.0)	.130	1.2 (0.8–1.8)	.334	1.0 (0.6–1.4)	.811
Continuous Use (*n* = 213)	48/24.2, 2.0 (1.4–2.5)	40/28.0, 1.4 (1.0–1.9)	39/27.6, 1.4 (1.0–1.9)	1.5 (1.0–2.3)	.049	1.4 (0.9–2.2)	.095	1.0 (0.6–1.5)	.894

^a^ Per woman-year since first report of ART use.

^b^ HRs were adjusted for the following baseline variables: presence of either *C. trachomatis* or *N. gonorrhoeae*.

^c^ Comparison group.

### Effect of contraceptive method on disease progression among women infected at enrollment (secondary effects)

Fifteen women were later found to be HIV infected at enrollment, thus randomized contraceptive use did not affect their primary HIV infection. Owing to the small number of women in this group (a maximum of 12 women per contraceptive comparison), we present these results descriptively. Among women infected at enrollment, we found that women randomized to DMPA-IM at the enrollment visit had higher mean VLs than those assigned copper IUD (5.66 vs. 3.83 log_10_ copies/mL; adjusted mean difference = 1.82, 95% CI: 0.73–2.91). There were no other statistically significant differences in VLs between methods at either the HIV detection visit or 3 months post-HIV detection. While we were not able to compare CD4 levels at the HIV detection visit (CD4 testing was not done at baseline), we found no statistically significant differences in mean CD4 levels between the three contraceptive groups at 3 months postdetection in either as-randomized or continuous use analyses. The number of events for both time to CD4 count <350 cells/mm^3^ and to viral suppression were too small to consider differences between groups for these analyses.

## Discussion

Contraceptive use is associated with decreased maternal–child mortality, reduced unintended pregnancy, and improved reproductive autonomy for women.^13^ We examined early HIV disease progression by contraceptive exposure among 382 HIV women who became HIV infected during the ECHO trial. We found that for women who became infected while using a contraceptive method, DMPA-IM use is unlikely to be associated with more rapid disease progression than the other studied methods. We found predictors of slower disease progression among women using DMPA-IM compared with copper IUD at the HIV detection visit, as evidenced by statistically significant lower mean VL and higher mean CD4 cell count among DMPA-IM users compared with copper IUD users. Among the minority of women who did not immediately initiate ART, this difference was not statistically significant at 3 months. We also found lower CD4 counts among copper IUD users than LNG implant users at the time of HIV detection in the continuous use analysis. Additionally, time to CD4 < 350 was somewhat shorter for IUD users than for DMPA-IM users and LNG implant users, whereas time to viral suppression was shorter in DMPA users than IUD users. These results should be interpreted with caution as several of the comparisons were not designated as primary outcomes *a priori*.

Our data do not allow for clear differentiation between potential primary and secondary effects of contraception on HIV disease progression. Among the 382 HIV-uninfected women at enrollment, differences at the HIV detection and subsequent visits may be attributable to a primary or secondary effect or both, as seroconversion occurred after contraceptive initiation. For those found to be HIV infected at enrollment (and thus before contraceptive initiation), any difference could only be attributed to a secondary effect. The number of women in this group (*n* = 15) is too small for meaningful statistical analyses but we report their outcomes to contribute data to future reviews on the effects of contraception on HIV progression. Thus, the implications from our results relate most directly to uninfected women at high risk of HIV infection who are starting a contraceptive method.

Some previous research, including a randomized trial and *in vitro* studies have suggested that some HC, particularly DMPA-IM, might result in more rapid HIV disease progression. For example, *in vitro* work suggests that MPA regulates expression of several genes involved in immune function and is consistent with MPA acting to increase both HIV-1 acquisition and pathogenesis, through mechanisms involving glucocorticoid-like effects on gene expression.^14^ A randomized trial among HIV-infected women in Zambia found that HC (including DMPA-IM and oral contraceptive) users had an increased risk of HIV disease progression (composite outcome of reduced CD4 count or death) compared with copper IUD users.^5^ That trial differs from most other studies in that it measured only secondary effects, and it is plausible that primary and secondary effects differ. In contrast, recent systematic reviews of multiple cohort studies as well as observational analyses from randomized trials have found no difference in HIV disease progression for HC users compared with women not using HC as measured by mortality, decreases in CD4 count, time to initiation of ART, and increases in VL. These include studies that specifically compared DMPA-IM users with women not using DMPA-IM or not using any HC.^6,7,15^ Additionally, a recently updated systematic review reports no evidence of faster HIV disease progression among copper-IUD users than users of other methods, primarily DMPA-IM and combined oral contraceptives.^16^

It is unclear why DMPA-IM use in this study was associated with somewhat slower disease progression compared with copper IUD use. It has been hypothesized that the overall effect of DMPA-IM on HIV acquisition may be a balance of both harmful (e.g., immune suppression) and protective effects (e.g., reduced viral exposure due to amenorrhea and/or reduced sexual activity).^17,18^ There was significantly reduced self-reported sexual activity after randomization to DMPA-IM compared with other groups in the overall ECHO study^10^ and lower sexual risk at seroconversion in the current cohort, including fewer reported coital acts and a lower prevalence of gonorrhea. Thus, it is feasible that lower exposure but greater susceptibility in the DMPA-IM group may have selected for viruses of lower fitness or virulence resulting in slower HIV disease progression for DMPA users that become infected.

The current analysis has a number of important strengths. Data come from a large, well-conducted randomized trial with high participant follow-up and for which HIV disease progression was a prespecified endpoint. Because we followed women who were initially HIV uninfected and followed them every 3 months, we were able to establish relatively accurate dates of HIV infection. Likewise, we were able to follow women from the point of seroconversion and thus evaluate the early effects of contraception on HIV disease progression. Our study had high rates of contraceptive continuation thus lending confidence to the as-randomized analysis. We were also able to conduct a continuous-use analysis that restricted follow-up time to those that continued to use their randomized method. The concordance of results from these two analyses provides confidence in our findings. Finally, we were able to examine disease progression measures before and after ART initiation—particularly important in this time of early ART initiation.

This analysis also has some limitations. Because we focused on HIV disease progression among women not on ART (and ART initiation was initiated promptly in most seroconverting participants), the amount of follow-up time between HIV detection and ART initiation was limited. Second, although we include an as-randomized analysis, a true intent-to-treat analysis was not possible as women were not randomized at the time of seroconversion and seroconverters made up only about 5% of the original randomized population. Thus, the characteristics of those infected may have differed between contraceptive groups before infection (i.e., selection bias) thus compromising the baseline comparability of the groups. For example, as DMPA is known to have immunosuppressive effects, CD4 may have differed by contraceptive groups before HIV infection. One previous study found no difference between HIV-uninfected women using DMPA, COCs, and not using hormonal contraception.^19^ However, no data exist comparing the CD4 cell levels of HIV-uninfected DMPA, copper IUD, and LNG implant users. Also, because only HIV-uninfected women were eligible for the trial, we have very few women who started contraception after HIV infection and hence limited ability to consider the separate secondary effects of contraception on HIV disease progression.

Access to safe and effective contraception for HIV-infected women is critical for reducing maternal and infant morbidity and mortality as well as for reducing the number of HIV-infected births. We found no evidence of accelerated HIV disease progression among DMPA-IM compared with copper IUD or LNG implant users but a suggestion, based on limited evidence that copper-IUD users were at greater risk of early disease progression compared with DMPA-IM and LNG implant users. This information is of relevance to women who need safe and effective contraception and are at high risk of acquiring HIV during contraceptive usage. Women at high risk of HIV should be provided a comprehensive package of HIV prevention methods and have regular testing to allow for prompt initiation of ART following HIV detection.

## ECHO Trial Consortium

### Management committee

Jared M. Baeten (University of Washington, Seattle, WA, USA), James Kiarie (WHO, Geneva, Switzerland), Timothy D. Mastro (FHI 360, Durham, NC, USA), Nelly R. Mugo (Kenya Medical Research Institute, Nairobi, Kenya & University of Washington, Seattle, WA, USA), and Helen Rees (Wits Reproductive Health and HIV Institute, Johannesburg, South Africa).

### Study site principal investigators

Eswatini, Manzini: Jessica Justman, Zelda Nhlabatsi (ICAP at Columbia University, New York, NY, USA and Family Life Association of Eswatini). Kenya, Kisumu: Elizabeth A. Bukusi, Maricianah Onono (Kenya Medical Research Institute, Nairobi, Kenya). South Africa, Brits: Cheryl Louw (Madibeng Center for Research). South Africa, Cape Town: Linda-Gail Bekker, Gonasagrie Nair (University of Cape Town & Desmond Tutu HIV Center). South Africa, Durban: Mags Beksinska, Jennifer Smit [MatCH Research Unit (MRU), Faculty of Health Sciences, University of the Witwatersrand]. South Africa, East London: G Justus Hofmeyr, Mandisa Singata-Madliki (University of Fort Hare and University of the Witwatersrand). South Africa, Edendale: Jennifer Smit [MatCH Research Unit (MRU), Faculty of Health Sciences, University of the Witwatersrand]. South Africa, Johannesburg: Thesla Palanee-Phillips (Wits Reproductive Health and HIV Institute, Faculty of Health Sciences, University of the Witwatersrand). South Africa, Klerksdorp: Raesibe Agnes Pearl Selepe (The Aurum Institute). South Africa, Ladysmith: Sydney Sibiya (Qhakaza Mbokodo Research Clinic). South Africa, Soshanguve: Khatija Ahmed (Setshaba Research Center). Zambia, Lusaka: Margaret Phiri Kasaro, and Jeffrey Stringer (UNC Global Projects Zambia and University of North Carolina at Chapel Hill, Chapel Hill, NC, USA).

### Other members of the ECHO Trial Consortium

Deborah Baron (Wits Reproductive Health and HIV Institute, Faculty of Health Sciences, University of the Witwatersrand, Johannesburg, South Africa), Deborah Donnell (University of Washington and Fred Hutchinson Cancer Research Center, Seattle, WA, USA), Peter B Gichangi (International Center for Reproductive Health—Kenya & Technical University of Mombasa, Mombasa, Kenya), Kate B Heller (University of Washington, Seattle, WA, USA), Nomthandazo Mbandazayo (Wits Reproductive Health and HIV Institute, Johannesburg, South Africa), Charles S Morrison (FHI 360, Durham, NC, USA), Kavita Nanda (FHI 360, Durham, NC, USA), Melanie Pleaner (Wits Reproductive Health and HIV Institute, Faculty of Health Sciences, University of the Witwatersrand, Johannesburg, South Africa), Caitlin W Scoville (University of Washington, Seattle, WA, USA), Kathleen Shears (FHI 360, Washington, DC, USA), Petrus S. Steyn (WHO, Geneva, Switzerland), Douglas Taylor (FHI 360, Durham, NC, USA), Katherine K. Thomas (University of Washington, Seattle, WA, USA), Julia D. Welch (FHI 360, Durham, NC, USA). Data management was done by DF/Net, Inc. (Seattle, USA); laboratory support was done by Clinical Laboratory Services (CLS), a division of the Wits Health Consortium (WHC) of the University of Witwatersrand's School of Pathology (Johannesburg, South Africa).
